# Menu Assessment Tools Used in Residential Aged Care: A Scoping Review of Content and Quality

**DOI:** 10.1111/jhn.70083

**Published:** 2025-07-21

**Authors:** Karly Bartrim, Alice Pashley, Mikaela Wheeler, Lily Chen, Lisa Sossen, Tracy Hancock, Danielle Cave

**Affiliations:** ^1^ School of Human Movement and Nutrition Sciences The University of Queensland St Lucia Queensland Australia; ^2^ Centre for Ageing Research and Translation University of Canberra Bruce Australian Capital Territory Australia; ^3^ School of Public Health The University of Queensland Herston Queensland; ^4^ Graduate School of Health University of Technology Sydney Ultimo New South Wales; ^5^ Lisa Sossen and Associates Elsternwick Victoria Australia; ^6^ School of Health University of the Sunshine Coast Sippy Downs Queensland Australia

**Keywords:** food and nutrition, menu assessment, residential aged care

## Abstract

This scoping review aimed to identify and synthesise existing publicly available tools that support menu assessments for residential aged care settings, specifically the tools content areas, recommendations and quality. The scoping review was conducted according to Levac et al. scoping review methodology and reported following the Preferred Reporting Items for Systematic Review and Meta‐Analyses extension for Scoping Reviews. Menu assessment tools were identified through grey literature through Google and Google Scholar search engines using key terms such as ‘residential aged care’ and ‘menu assessment’. Data were synthesised and reported narratively, according to content areas of menu planning, nutritional basis of the menu, texture modification/fluid consistency, therapeutic and special diets and foodservice management recommendations. Quality appraisal was conducted using the Appraisal of Guidelines for REsearch & Evaluation Instrument. Seventeen tools from seven countries were included. There was variability in the content and recommendations, with no included tool covering all content areas. The overall quality ranged from two to six (out of seven), and no tools met the quality criteria to be recommended for use in their current form. The lack of consistency in content areas, recommendations and quality of tools indicates the need to modify existing tools or to conduct research to support the development of a new evidence‐based tool. Future research is needed to gain consensus on acceptable content areas, appropriate recommendations and to develop an evidence‐base to underpin the tools. A purpose‐built, evidence‐based tool is essential for consistent foodservice evaluation and for improving food and nutrition standards, ultimately enhancing resident outcomes.

## Summary

- A diverse range of menu assessment tools exist internationally that have large variation in content, level of detail and guidance provided, and quality.
- The review highlights that future research is required to seek international consensus on acceptable content areas and recommendations included in menu assessment tools.
- Nationally, in Australia there is a need to develop a comprehensive, evidence‐based menu assessment tool with resident and stakeholder input to support the consistency of dietitian practice.

## 1 Introduction

Food and nutrition play an important role in maintaining health and quality of life (QoL) for older adults in residential aged care (RAC) [1, 2, 3]. For the majority of older adults living in RAC (also known as long‐term care, nursing homes or care homes), food is primarily provided and controlled by the home. Therefore, RAC foodservices need to ensure that high‐quality food is provided that meets residents’ preferences and nutritional, psychosocial and cultural needs [2]. Additionally, mealtimes in RAC are an important social activity, supporting normality and comfort [4, 5, 6, 7]. Despite the known benefits of good nutrition and food provision, opportunities exist to improve the quality of meals and menus provided to residents in RAC [1, 2, 7, 8, 9, 10]. In particular, improvements that increase choice [11], food quality [7], variety [7, 12] and overall nutritional value persist [2, 12]. Improving the quality of food delivered in RAC is economically justified [13, 14], given its contribution to reducing the high malnutrition prevalence of up to 50% of residents across RACs [15, 16].

Menus are the fundamental control of foodservice activity, which can affect resident satisfaction, food intake, staff workload and financial outcomes [5]. A planned and well‐considered menu provides a foundation for nutrition interventions required for individual resident care [5]. Menu planning and assessments are considered essential components of RAC food and nutrition standards [5] and guidelines [5, 17]. Menu planning requires consideration of the residents’ preferences and nutritional needs, food production practicalities and foodservice logistics to support residents’ health, nutritional status and QoL [5, 9]. Menu assessments can involve reviewing the day‐to‐day operations of the food system and exploring food provision to residents, including nutritional quality, variety, choice and alignment with cultural or religious needs [10, 18, 19]. For the purpose of this review, the term menu assessments will be used to describe both the planning and assessing of menus.

Globally, there is variation in how menu assessments are conducted, with legislation playing a role in shaping both their scope and implementation [10, 17, 20, 21]. For example, in Canada, menu assessment requirements vary in each province and there are also differences in nutrition requirements (i.e., the menu must comply with Canada's Food Guide or take into consideration the Dietary Reference Intakes of residents) [10, 20]. Additionally, variation also exist with who plans menus (i.e. dietitians and/or nutrition managers), with one province outlining the requirement of menu being signed off by a dietitian [10, 20]. These specific menu assessment requirements are vastly different to the United Kingdom, where currently there are no specified requirements or legislation requiring a menu assessment [17]. In Australia, the new Strengthened Aged Care Quality Standards (Australian Quality Standards) come into effect in 2025, which will mandate that all RACs are required to have input from an Accredited Practising Dietitian in menu development and complete a menu assessment at least annually [21]. Despite this new requirement in Australia, no nationally endorsed menu assessment tool (MATs) exists for RAC. Additionally, tools that exist internationally to conduct a menu assessment have not been explored.

With variations in menu assessments globally, clarifying ideal approaches to inform future practice may be beneficial. While a recent scoping report [22] has provided the first insight into MATs that exist, the paper focused on whether the information in the tools reflects the recommendations outlined in the Australian Quality Standards [21]. As such, the content and recommendations included in the tools, and the quality of the evidence that underpins them is still unclear [22]. Understanding the content, recommendations and quality of publicly available MATs is imperative to identify opportunities to improve menu assessments in RAC internationally and enhance food and nutrition for residents living in RAC. Therefore, this scoping review aims to identify and synthesise existing publicly available tools that support menu assessments for RAC settings, specifically the tools content areas, recommendations and quality.

## 2 Materials and Methods

### 2.1 Study Design

A scoping review was conducted to identify the key characteristics and quality of MATs designed for RACs [23]. This scoping review was conducted according to Levac et al. methodology for scoping reviews, which follows five steps: (1) identify the research questions, (2) identify relevant studies, (3) study selection, (4) charting the data, and (5) collating, summarising and reporting the results [24, 25]. The Preferred Reporting Items for Systematic Review and Meta‐Analyses extension for Scoping Reviews (PRISMA‐ScR) was followed in the reporting of this manuscript [26]. A protocol was not registered for this review.

### 2.2 Search Strategy

A search of the grey literature was conducted using Google and Google Scholar search engines. This review focused on publicly available tools to assess what is available to the dietitians who conduct these reviews. The search terms used for Google Scholar were: concept 1—‘aged care’ OR ‘residential care’ OR ‘long term care’ OR ‘long‐term care’ OR ‘nursing home’ OR ‘elderly care’ OR ‘care homes’ OR ‘older adult’ OR ‘institution’ AND concept 2—‘menu review’ OR ‘menu assessment’ OR ‘menu standard*’ OR ‘menu guideline’ OR ‘menu checklist’ OR ‘menu development’ OR ‘menu recommendation*’ OR ‘menu design’ OR ‘menu criteria’ OR ‘meal criteria’ OR ‘food standard’ OR ‘food guideline’. The search terms used for the Google search included 108 combinations of the concept 1 and concept 2 terms. The searches were carried out between January 28 and February 21, 2024, with an additional search conducted between January 28 and February 16, 2025, to capture any updated or newly published tools. The first 100 results were searched. Searches were conducted using the Google Chrome browser in incognito mode for private browsing. The decision to focus on grey literature only was due to grey literature being the primary source for MATs, and a preliminary search of academic databases yielding no relevant results. All records were screened by their title and either the supplied summary (Google) or the abstract (Google Scholar). The reference lists of the included tools were also reviewed to identify other relevant tools.

### 2.3 Eligibility Criteria

Documents were included if they met the following criteria: (1) were developed for RAC or international equivalent care setting for adults ≥ 65 years (e.g., care home, nursing home, long‐term care), (2) publicly available in full text, (3) published in English, (4) had not been superseded by another version, and (5) provided guidance on how to assess a menu in any document format, for example, checklist, guidelines or standards. Documents were excluded if they focused on the community care setting or had limited information regarding menu planning (i.e., one or less menu assessment content area). There were no date restrictions.

### 2.4 Data Extraction

Data were extracted into content areas, which were identified by the team of dietitian researchers who have published research on nutrition and foodservice topics in RAC. Data were extracted in duplicate into an Excel [27] spreadsheet by two independent researchers to ensure the accuracy and completeness of the data. If any discrepancies arose, an opinion of a third researcher from the broader research team was sought or it was discussed at the bi‐monthly team meetings. For a content area or item to be included, it had to be of relevance (i.e., reported in more than one tool), and consensus had to be reached by the majority of authors (~80%). Data were extracted from the tools, including title, author(s), publication year, setting/population, type of tool, occupation of the authors who developed the tool and who published the tool, for example, government or organisation. Data were extracted verbatim under content areas of:

1. Menu planning—recommendation for stakeholder involvement in menu assessment, resident input into menu planning/menu assessment, recommended menu change, mealtime and food offering, choice, variety, portion and serve/serving size and standardised recipes.
2. Nutritional targets—reference person, food groups, energy and protein target, macronutrients, fibre, fluid, micronutrients and food fortification for energy and protein.
3. Texture modification/fluid consistency/therapeutic and special diets—texture modification/fluid consistency, therapeutic modification, finger food, cultural and religious recommendations, vegetarian/vegan and special occasions and celebrations. Therapeutic modification was defined as altered macro‐ or micronutrient diets for health purposes (e.g., allergies, intolerances, medical conditions such as diabetes).
4. Foodservice management recommendations—frequency of menu review, budget/cost recommendations, food safety, dining experience/environment, menu availability, presentation and details for residents, sample menu or recipes, software analysis, staff training and sustainability considerations.

### 2.5 Data Synthesis

Results were synthesised and reported narratively, according to the content areas. Descriptive statistics (i.e., count and percentage) were used to summarise the number of MATs that discussed the content area. Content areas and recommendations were reported verbatim or were summarised in relation to their degree of specificity. For example, for choice, recommendations were categorised as (1) general recommendation to provide choice with no specific targets, (2) recommendations for the level of choice to be provided for some aspects (e.g., per meal or food group), and (3) specific recommendations for the level of choice to be provided (e.g., explicitly refers to choice provided each day and/or across the menu). For brevity, all included guidelines, tools, and checklists are referred to as tools in the results.

### 2.6 Quality Appraisal

Quality appraisal was conducted using the Appraisal of Guidelines for REsearch & Evaluation Instrument (AGREE II), which assesses methodological rigour and transparency of guideline or tool development [28]. The tool contains 23 items rated on a 7‐point scale (1 = strongly disagree; 7 = strongly agree) and are organised into six domains: (1) Scope and Purpose, (2) Stakeholder Involvement, (3) Rigour of Development, (4) Clarity and Presentation, (5) Application, and (6) Editorial Independence. The AGREE II tool also has an overall score for the quality of the tool [28]. Quality appraisal was completed by four independent researchers, as recommended [28], to increase the reliability of the assessment. Researchers also met as a group and discussed their assessments quarterly throughout the process. Domain scores were calculated by totalling the scores of individual items (in each domain) across all reviewers and scaling the total as a percentage of the maximum possible score for that domain [28]. The scaled domain score was calculated using the following formula [28]:

$$\frac{\text{obtained}\;\text{score}‐\text{minimum}\;\text{possible}\;\text{score}}{\text{maximum}\;\text{possible}\;\text{score}‐\text{minimum}\;\text{possible}\;\text{score}}.$$

Domain scores are presented as a percentage, with 50% set as the benchmark for meeting each domain criteria, which is consistent with thresholds used in previous studies [29, 30]. Tools that scored > 50% in all domains and scored four or more (out of seven) for overall quality, were recommended for use (yes). Tools that scored > 50% in four or more domains and scored four or more for overall quality, were recommended for use with modifications (yes, with modifications). Tools that scored < 50% in three or more domains and scored three or less for overall quality were not recommended for use (no).

## 3 Results

**Figure 1:**
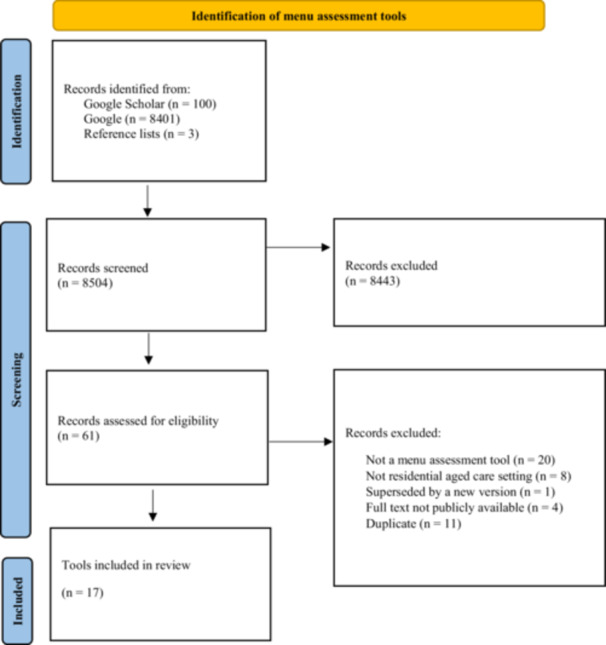
Flow chart of study selection of menu assessment tools.

A total of 8504 records were identified (see Figure 1 for tool selection). The full texts of 61 records were assessed for eligibility. Of the 61 records assessed, a total of 44 records were excluded, with reasons for exclusion including not a MAT (*n* = 20), not RAC setting (*n* = 8), superseded by a new version (*n* = 1), full text not publicly available (*n* = 4) or duplicate (*n* = 11). A total of 17 MATs were included in this review.

Six of the included tools were developed in Australia, with five tools designed for use in Australian states of Queensland, New South Wales, Victoria, South Australia and Western Australia [31, 32, 33, 34, 35] and a tool developed for a local council area of Cape York in North Queensland [36]. Four tools were developed in Canada, with two specific to the province of Ontario [5, 20, 37, 38], three in England [39, 40, 41], one in Wales [42], one state‐based tool for Maryland in the United States [43], one in Northern Ireland [44] and one in the United Kingdom [17]. Tools were released between 2004 and 2024, with seven released in the past 5 years. Most tools (*n* = 13, 76%) had a sole focus on RAC [5, 17, 20, 33, 36, 37, 38, 39, 40, 41, 42, 43, 44]. Four tools had a hospital focus with a subsection on RAC [31, 32, 34, 35]. The type of documents included differed in design type or purpose, with some described as ‘standards’ [5, 31, 32, 34, 35], ‘manuals’ [33, 38, 43], ‘toolkits’ [36, 39], ‘guidance documents’ [37, 40], ‘guidelines’ [17, 41], checklist’ [44] and ‘working paper’ [20]. Of the included tools in the review, seven tools were developed by dietitians [5, 17, 20, 37, 44] or a ‘nutrition team’ [36, 39], five tools were developed with a range of professionals (i.e., managers, speech pathologists, occupational therapists, dentists, researchers and RAC staff) including a dietitian [32, 33, 34, 41, 42] and five tools did not specify occupation or reference authors of the tool [31, 35, 38, 40, 43]. Only two tools engaged a consumer in the tool development [32, 34]. The tools were published by State/Provincial Governments (*n* = 7, 41%) [31, 32, 33, 34, 38, 43], National Governments (*n* = 3, 18%) [40, 42, 44], professional organisations (i.e., dietetic association) (*n* = 4, 24%) [5, 17, 20, 37], a health organisation (*n* = 1, 6%) [36] and a trust (*n* = 1, 6%) [41]. No tools reported that they were pilot tested or tested for feasibility, efficacy or effectiveness.

### 3.1 Menu Planning and Assessment Recommendations

Information relating to menu planning and assessment recommendations are presented in Table 1. More than half of tools (*n* = 11, 65%) provided recommendations for stakeholder involvement in menu planning and/or menu assessments [5, 17, 20, 31, 32, 33, 34, 35, 38, 39, 44]. Stakeholders recommended for involvement included dietitians (*n* = 8, 47%) [5, 17, 20, 31, 32, 33, 38, 39], the residents and/or their families (*n* = 8, 47%) [5, 17, 20, 31, 32, 34, 38, 39], foodservice managers (*n* = 6, 35%) [17, 31, 32, 34, 38, 39], with one (6%) tool recommending including a speech pathologist [34]. While residents are engaged in the menu assessment process, only eight (47%) provided examples of how to collect and incorporate resident input [17, 20, 35, 36, 37, 38, 41, 42].

**Table 1:** Information reported in the menu assessment tools relating to menu planning and assessment.

Title of document, organisation/author, year of publication and reference	Setting	Recommendation for stakeholder involvement in menu planning and/or menu assessment	Resident input into menu planning/menu assessment	Menu cycle length	Recommended menu change	Mealtime and food offerings	Choice	Variety	Portion and serve sizes^a^	Standardised recipes
*Australia*										
Nutrition Standards for Meals and Menus, Queensland Health, 2022 [31]	Hospital, with RAC component.	Dietitians, foodservice managers, other key stakeholders, residents and their families.	Specifies residents’ food and fluid preferences should be considered in menu planning and residents should have input into menu choices and menu reviews. However, no specific recommendations on getting resident input.	≥ 2 weeks.	—	Main meals and mid‐meals.	Provides specific recommendations for the level of choice to be provided.	Provides specific recommendations for menu variety.	Serve size for meals (small, medium and large), and portion size for meal components.	—
						Additional food and/or fluid supplements shall be available as required including accessible snack options any time of the day and night.				
Apunipima Remote Residential Aged Care Menu Assessment Toolkit, Apunipima Cape York Health Council, 2015 [36]	Remote RAC (& community) across 11 communities in Cape York.	—	Specific recommendation‐ meal satisfaction survey could be used.	≥ 2 weeks.	—	Main meals and mid‐meals.	—	Provides specific recommendations for menu variety.	Serve size for meal components (according to AGHE).	—
Nutrition and Quality Food Standards for Adults in Victorian Public Hospitals and Residential Aged Care Services, Victorian Government Department of Health & Alfred Health Nutrition Department, 2022 [32]	Hospital, with RAC component.	Foodservice dietitian, foodservice manager, residents and their families.	Specifies documenting residents’ menu and meal preferences, no specific recommendations on getting resident input.	1 week minimum.	—	Baseline regular texture and TM diets provide three main meals and three snacks per day.	Provides specific recommendations for the level of choice to be provided.	Provides specific recommendations for menu variety.	Serve size for meal size and meal components.	All menu items have documented standardised recipes and/or product specifications with serve sizes that have been endorsed by a foodservice dietitian and are followed by chefs/cooks and foodservice staff.
						Band 1 snacks be made available 24 h daily and offered after physical therapy/exercise.				
Best Practice Food and Nutrition Manual for Aged Care Homes, New South Wales Government, 2015 [33]	RAC	Dietitians to consults residents, family and staff.	Specifies consulting residents on their preferences and resident favourite dishes are included.	4 weeks.	—	Main meals, mid‐meals and ‘24‐hour dining’, residents can access meals as well as snacks around the clock.	Provides recommendations for the level of choice to be provided at some meals.	Provides general recommendations for menu variety.	Portion size for soups and serve size for desserts.	Mentions standardising recipes as a quality improvement project.
			However, no specific recommendations on getting resident input.							
Menu and Nutritional Standards for Public Health Facilities in South Australia, Department for Health and Wellbeing (Government of South Australia 2020) [34]	Hospital, with RAC component.	Dietitians, foodservice managers, consumers and other key stakeholders. Speech pathologists should be consulted.	Specifies residents food and fluid preferences should be considered in menu planning and residents should have input into menu choices and menu reviews.	4 weeks.	—	Main meals, mid meals and additional food and fluids should be available as required.	Provides specific recommendations for the level of choice to be provided.	Provides specific recommendations for menu variety.	Serve sizes for meal components and meal size.	Requires ‘Standard Recipes’ to be in place.
			However, no specific recommendations on getting resident input.							
Nutrition Standards for Adult Inpatients and Residential Aged Care Policy, Government of Western Australia, Country Health Service, 2020 [35]	Hospital, with RAC component.	Dietitians, catering services, speech pathologists and operation managers.	Specific recommendation— Customer Satisfaction Survey —should be done by all sites at least annually regardless of the number of beds. This includes questions on menu popularity.	≥ 2 weeks.	—	—	Provides specific recommendations for the level of choice to be provided.	Provides general recommendations for menu variety.	Portion sizes for meal components, meal size and serve sizes for meal components.	—
*United Kingdom*										
England										
Guidance on Food Served to Older People in Residential Care, Food Standards Agency, 2007 [40]	RAC.	—	—	—	—	Main meals and mid meal snacks.	—	Provides general recommendations for menu variety.	Portion size of meal components.	—
Eating Well for Older People, The Caroline Walker Trust, 2004 [41]	RAC.	—	Specific recommendation— Wherever possible, residents should be encouraged to contribute their own recipes.	5 weeks	Every 3 months	Main meals and snacks should be provided in between more formal mealtimes.	—	Provides general recommendations for menu variety.	Portion sizes for meal components and meal size.	—
Healthier and More Sustainable Catering. A Toolkit for Serving Food to Older People in Residential Care, Public Health England, 2017 [39]	RAC.	Residents, procurement managers, catering managers.	—	—	—	Main meal and be snacks.	—	Provides general recommendations for menu variety.	Serve size and portion sizes for meal components.	States ‘check recipe sheet’, however no specific mention or recommendations for standardised recipes.
Care Home Digest, Menu planning and food service guidelines for older adults living in care homes, British Dietetic Association Food Services Specialist Group and the Older People Specialist Group, 2024 [17]	RAC.	Dietitian, chef, catering teams, managers, nursing staff, carers and nutrition champions.	Specifies importance of ensuring residents’ preferences are built into menus using resident feedback and knowledge of the most popular menu choices. Specific recommendations‐ Feedback could be in the form of: a suggestion book made available in the dining room, feedback cards to capture how the meal was today, regular resident/relative meetings or food forums, surveys for residents, friends and families and observations and waste records can be helpful (if resident unable or does not want to verbalise their views).	3–4 weeks	—	Breakfast, main meal, simple (second) meal and snacks. No more than 5 h between each meal, and overnight no more than 12 h from evening snack to breakfast. Food should also be available 24 h per day for those who may be awake during the night.	Provides specific recommendations for the level of choice to be provided.	Provides general recommendations for menu variety.	Portion size for meal components and meal size.	Refers to a recipe management system and a recipe database system. No specific mention of standardised recipes.
Northern Ireland										
Nutritional guidelines and menu checklist for residential and nursing homes, Public Health Agency, 2014 [44]	RAC.	Residents.	Specifies importance that menus should take into account residents’ preferences. However, no specific recommendations on getting resident input.	—	Regularly, no specific timeframe recommended.	Three full meals and snacks should be served every day at regular intervals (no more than 5 h intervals), of which at least one meal should be a cooked choice. The interval between the evening snack and breakfast the following morning should not be more than 12 h.	Provides specific recommendations for the level of choice to be provided.	Provides general recommendations for menu variety.	Serve sizes provided for each food group.	—
Wales										
Food and Nutrition in Care Homes for Older People, Welsh Government, 2019 [42]	RAC.	—	Specifies importance of talking to residents and relatives when planning menus.	≥ 3 weeks.	Twice per year and seasonality is considered.	Three main meals and snacks.	—	Provides general recommendations for menu variety.	Portion sizes for meal components and meal size.	Standardised recipes are used to ensure consistent quality and content.
			Specific recommendation‐ Have conversations with individual residents and/or relatives and friends, about their own food stories and what food means to them. Have recipe sharing sessions and ask the cooks to try them out.			A range of snacks are available to the residents, 24 h a day. Intervals between meals do not exceed 5 h and the interval between the last meal offered and breakfast the following morning is less than 12 h.				
*United States of America*										
Diet Manual for Long Term Care Residents, The Office of Health Care Quality, 2014 [43]	RAC.	—	—	—	—	Three balanced meals and up to three snacks.	—	—	Portion sizes for meal size (small, medium and large).	Refers to standardised recipes for different diets, however, does not explicitly mention.
*Canada*										
Best Practices for Nutrition, Food Service and Dining in Long Term Care Homes, Ontario Long Term Care Action Group, 2019 [20]	RAC.	Registered Dietitian, residents, family members, substitute decision makers, other designated parties, and appropriate team members.	Specifies importance of residents’ preferences and appetites are routinely assessed.	3–4 weeks.	Annually, with adjustments made seasonally (Spring/Fall).	A minimum of three meals, three additional beverage opportunity passes and two snacks daily. In addition, food and beverages are available for residents on a 24‐h basis.	—	Provides general recommendations for menu variety.	—	Standardised recipes and portion sizes are developed and used consistently for each menu item. This includes all foods and fluids that are modified in texture or consistency.
			Specific recommendation—A residents’ food committee can be established for planning and approving cycle menus and special occasion menus. Also, residents’ satisfaction questionnaires and Residents’ Council and/or Food Committee comments.							
Menu Planning in Long Term Care, Dietitians of Canada, 2020 [37]	RAC.	—	Specifies importance that residents are offered choice. Prepared/processed food options are commonly requested and/or are popular with residents, and inclusion on the menu should be based on resident input. Specific recommendation‐ documented approach to ensure resident input into menu planning process (e.g., by review of meeting minutes from the Resident Council and/or Resident Food Committee).	≥ 3weeks.	At least annually, with updates seasonally.	Three meals and minimum of two snacks (afternoon, evening) and three between‐meal beverages (morning, afternoon, evening). Morning snacks added based on resident needs and preferences and timing of meals.	Provides recommendations for the level of choice to be provided at some meals.	Provides general recommendations for menu variety.	Portion sizes for meal components and meal size.	Standardised recipes for palatable and acceptable nutrient‐dense foods with reasonable costs are needed.
National Long Term Care Standards Project: Recommended Food & Nutrition Standards (Background Document), Dietitians of Canada (Brauer, P, Mardinlin‐Vandewalle L & Whittington‐Carter L), 2022 [5]	RAC.	Registered Dietitian, residents, family members, substitute decision makers, other designated parties, and appropriate team members.	Specifies importance of nutrient‐dense foods tailored to residents’ needs and preferences. However, no specific recommendations on getting resident input.	3‐4 weeks.	On a regular basis, at least annually.	The master menu includes a minimum of three meals, three additional beverage opportunity passes and two snacks daily.	Provides recommendations for the level of choice to be provided at some meals.	Provides general recommendations for menu variety.	—	Standardised recipes and portion sizes are developed and used consistently for palatable and acceptable nutrient‐dense foods, including food and fluids modified in texture or consistency.
						Food and beverages appropriate to residents’ diet and texture requirements are available for residents on a 24‐h basis or outside of regular mealtimes.				
Audits and More: A Nutrition and Food Service Audit Manual for Adult Residential Care Facilities with 25 or more Persons with Care, Government of British Columbia, 2008 [38]	RAC.	Registered dietitian, foodservices/nutrition manager, residents.	Specific recommendation‐ consultation process used, which includes satisfaction questionnaires and food committee comments.	≥ 4 weeks.	Minimum twice annually.	Three meals and at least 2 snacks offered each day (one snack is offered in the evening). Beverages available at all meals and snacks include water, juice and milk in addition to coffee and tea.	Provides general recommendation to provide choice.	Provides general recommendations for menu variety.	Portion sizes for meal components and meal size.	Standardised recipes available for all types of food items.
										In order for the analysis to be accurate, the menu must be supported by standardised recipes. Also mentioned other benefits of standardised recipes.

Abbreviation: AGHE = Australian Guide to Healthy Eating.

^a^ Portion size refers to the amount of food a resident may choose to eat at one time, whereas serve/serving size refers to the amount of food that the resident is served at one time. Tools mentioned portion size, serve size, both or used the terms interchangeably to describe recommendations for meal components and/or meal size.

Menu cycle length was mentioned in 12 (71%) tools. The most commonly (*n* = 7, 41%) recommended menu cycle length was recommended between 3 and 4 weeks [5, 20, 33, 34, 37, 38, 42], with three (18%) tools recommending > 2 weeks [31, 35, 36], one (6%) recommending 1 week minimum [32], and one recommending 5 weeks [41]. Menus were recommended to be changed in seven (41%) tools, with one tool suggesting every 3 months [41], two tools every 6 months [38, 42], three tools suggesting at least annually [5, 20, 37] (with two specifying seasonal [Spring/Fall] adjustments) [20, 37] and one tool recommended menu changes but specified no time frame [44]. With mealtime and food offerings, 15 tools (88%) specifically mentioned main meals, mid meals and/or snacks [5, 17, 20, 31, 32, 33, 34, 36, 37, 38, 39, 40, 41, 42, 44], with eight tools (47%) describing the need to provide residents with 24‐h access to food or fluid [5, 17, 20, 31, 32, 33, 34, 42].

Recommendations for choice were provided in 10 (59%) tools, with seven tools (41%) providing specific recommendations for choice [17, 31, 32, 33, 34, 35, 44], two tools recommended choice at some mealtimes [5, 37, 38] and one tool recommended to offer choice but did not specify how [37]. Recommendations for variety were provided in 16 tools (94%), with 12 tools (71%) providing recommendations for menu variety however did not provide a definition or details on how to provide variety (e.g., mentioned offering a variety of vegetables at main meals) [5, 17, 20, 33, 35, 37, 38, 39, 40, 41, 42, 44], and four tools provided specific recommendations [31, 32, 34, 36]. Tools mentioned portion size, serve size, both or used the terms interchangeably to describe recommendations for meal components and/or meal size. Seven tools referred to portion size for meal components [17, 31, 35, 37, 38, 40, 42], four tools referred to serve size for meal components [32, 34, 35, 41] and two tools used the terms interchangeably [33, 39]. For meal size, five tools used the term portion size [17, 37, 38, 42, 43] and seven tools referred to serve size [31, 32, 34, 35, 36, 41, 44]. Seven tools (41%) referred to standardised recipes [5, 20, 32, 33, 34, 35, 42], with only three (18%) explicitly mentioning the need for standardised recipes [17, 39, 43].

### 3.2 Nutritional Targets for Menus

An overview of the nutritional basis of each tool is presented in Table 2. Twelve tools (71%) used both food groups and energy and protein targets as the nutritional basis of their menu [20, 31, 33, 35, 37, 39, 40, 41, 42, 43], three tools used only energy and protein targets [5, 31, 34] and two tools used food groups [36, 44]. Of the tools that used energy and protein targets, eight (47%) provided a reference person for their calculations based on specific demographics (i.e., sex, age, height, and/or weight) [31, 32, 33, 34, 35, 39, 41]. Two tools recommended using a reference person, but did not specify one or provide information on how to identify a reference person [38, 40], and no tools recommended the same demographics for a reference person. More than half of the tools (*n* = 10, 59%) that referred to food groups were based on their respective national guidelines [20, 32, 33, 35, 36, 38, 39, 41, 42, 43, 44] with only two tools adapted guidelines for the RAC population [37, 40], one referred to ‘food groups’ [17] and one acknowledged challenges of using national guidelines for the RAC population [33].

**Table 2:** Information reported in menu assessment tools relating to nutritional targets.

Title of document and reference	Basis of menu—nutritional reference			Other nutrients				Food fortification
	Reference person	Food groups	Energy and protein target^a^	Macronutrients	Fibre	Fluid	Micronutrients^b^	Energy and protein
*Australia*								
Nutrition Standards for Meals and Menus [31]	Male ≥ 70 years with average weight 76 kg.	—	Energy: 105–125 kJ/kg/day. Protein: 1.2–1.6 g/kg/day.	—	Fibre: 25–30 g/day.	—	Omega 3: fish 3 times/week.	Fortify hot cereal and soups.
								Nutrient targets—hot cereal: ≥ 550 kJ & ≥ 10 g per serve
								Soup: Group 1— ≥ 600 kJ & ≥ 8 g per serve.
								Group 2—≥ 400 kJ & ≥ 5 g per serve.
Apunipima Remote Residential Aged Care Menu Assessment Toolkit [36]	—	AGHE.	—	—	—	—	—	—
Nutrition and Quality Food Standards for Adults in Victorian Public Hospitals and Residential Aged Care Services [32]	Male ≥ 85 years, 68 kg, 165.4 cm, 24.9 kg/m^2^.	Must align with Australian Dietary Guidelines, no specific reference amounts.	Energy: 125 kJ/kg/day. Protein: 1.5 g/kg/day (minimum)	Fats: 20%–35% total daily energy (< 10% saturated/trans fats). Carbohydrates: 45%–65% total daily energy.	Fibre: 30 g/day.	Fluid: adequate intake of 2.6 L/d.	Sodium: (suggest dietary target)‐ 2000 mg/day, calcium: 1000–1300 mg/day, iron: 8–18 mg/day, zinc: 14 mg/day, vit C: 45 mg/day, folate: 400 μg/day.	Fortify mashed potato, milk, vegetables, soups and desserts.
			Or a suggestion that the baseline menu should provide a minimum: Energy: 8500 kJ. Protein: 85 g					No nutrient targets.
Best Practice Food and Nutrition Manual for Aged Care Homes [33]	Male and females (sedentary) ≥ 71 years, BMI 22–27 kg/m^2^.	Acknowledged ADG, however also makes references to the challenges of application in RACF setting.	Energy: 125–146 kJ/kg/day. Protein: 1.5–3 g/kg/day.	—	Fibre: 25–30 g/day.	Fluid: minimum 1.6–2 L/day.	l‐Arginine: 9 g/day (for wound healing).	Fortify mashed potato, milk, vegetables, soups and desserts.
							Calcium: 1300 mg/day	No nutrient targets.
							Provides a checklist for all 2005 RDI or AI for sedentary males/females 71 years and older.	
Menu and Nutritional Standards for Public Health Facilities in South Australia [34]	Male: weighing 76 kg, and female weighing 65 kg (consistent with NRVs for male ( ≥ 70 years) and female (19‐50 years).	—	Energy: 8000–9500 kJ/day. Protein: 90 g/day or > 1.2 g/kg/day.	Fat: menu items should not routinely be low in total fat.	Fibre: 30 g/day.	Fluid: 2.1–2.6 L/day.	Folate: 400μg/day, calcium: 1300 mg/day, iron: 13 mg/day, zinc: 14 mg/day. sodium: intake limit.	Fortify hot cereal, soup, mashed potato and milk.
								No nutrient targets.
Nutrition Standards for Adult Inpatients and Residential Aged Care Policy, Government of Western Australia [35]	Male: weighing 76 kg male aged 51–70 years.	Recommended number of serves from Core Food Groups.	Energy: 8000–9500 kJ/day (105–125 kJ/kg/day). Protein: 90 g/day (1.2 g/kg).	Fat: < 10% energy from trans and saturated fat.	Fibre: 30 g/day.	Fluid: 2.1‐2.6 L/day.	Vit C: 45 mg/day, folate: 400 μg/day, calcium:1000 mg/day, iron: 11 mg/day, zinc: 14 mg/day and sodium: < 2000 mg/day	Fortify hot cereal, soup, sauces, gravies, wet dishes, mashed vegetables.
								No nutrient targets.
*United Kingdom*								
England								
Guidance on Food Served to Older People in Residential Care [40]	Must meet average population requirement of RACF. Suggestions in document are for ≥ 75 years.	Food based guidelines modified based on government advice‐ Food Standards Agency.	Energy: 1955 kcal/day (8170 kJ). Protein: 50 g/day.	Fat: 76 g/day max (saturated fat 24 g/day max), carbohydrate: 260 g/day minimum.	Fibre: 18 g/day.	Fluid: 1.2 L/day.	Vitamin D: 10 μg/day (minimum), folate: 200 μg/day (minimum), iron: min 9 mg/day (minimum), zinc: min 9.5 mg/day (minimum), Sodium: 2400 mg/day (maximum), salt: 6 g/day (maximum), potassium: 3500 mg/day (minimum), magnesium: 300 mg/day (minimum) and riboflavin: 1.3 mg/day (minimum).	—
Eating Well for Older People [41]	≥ 75 years.	Committee on Medical Aspects of Food and Nutrition Policy (COMA).	Women‐Energy: 7566 kJ/day (1810 kcal/day).	Fat: 35% of diet (women > 75 years 70 g/day, men > 75 years 82 g/day), carbohydrates: 50% of food energy (39% starch, 11% sugars).	Fibre: 18 g/day.	Fluids: 1.5 L/day.	Folate: 200 μg/day, vit C: 40 mg/day, vitamin A: 600–700μg/day, vitamin D: 10 μg/day, calcium: 700 mg/day, iron: 8.7 mg/day, zinc: 7‐9.5 mg/day, potassium: 350 mg/day, sodium: < 2400 mg/day, thiamin: 0.8–0.9 mg/day, riboflavin: 1.1–1.3 mg/day, niacin: 12–16 mg/day.	Fortify milk, drinks and desserts.
			Protein: 46.5 g/day.					No nutrient targets.
			Men‐ Energy: 8778 kJ/day (2100 kcal/day).					
			Protein: 53.3 g/day.					
Healthier and More Sustainable Catering. A Toolkit for Serving Food to Older People in Residential Care [39]	≥ 75 years.	Committee on Medical Aspects of Food and Nutrition Policy (COMA).	Energy: 7892 kJ/day (1888 kcal/day).	Total fat: 73.4 g/day (saturated fat 23.1 g/day), Carbohydrates: 252 g/day.	Fibre: 30 g/day.	Fluid: 6–8 cups or glasses/day.	Vitamin A: 700 µg/day, thiamin: 0.9 mg/day, Riboflavin: 1.3 mg/day, Niacin equivalent: 15.1 mg/day, vitamin B6: 1.4 mg/day, vitamin B_12_: 1.5 µg/day, folate: 200 µg/day, vitamin C: 40 mg/day, vitamin D: 10 µg/day, calcium: 700 mg/day, magnesium: 300 mg/day, potassium: 3500 mg/day, iron: 8.7 mg/day, zinc: 9.5 mg/day, copper: 1.2 mg/day, selenium: 75 µg/day, iodine: 140 µg/day.	—
			Protein: 50 g/day (minimum).					
Care Home Digest, Menu planning and food service guidelines for older adults living in care homes [17]	—	Refers to food groups and the amount of each food group to provide per meal.	Energy: 8360 kJ/day (2000 kcal/day). Protein: 75 g/day.	Fat: food swap recommendations only.	—	Fluid: Women: 1.6 L/day and Men: 2 L/day	—	Fortify drinks (milk and juice) and dishes, provides examples of nutrient‐dense food fortification ingredients with suggested portion sizes.
				Carbohydrates: per meal recommendations (i.e., between 120–180 g per main meal and specific breakfast recommendations).				
								Nutrient target: Increase energy by ≥ 2092 kJ (500 kcal) per day.
Northern Ireland								
Nutritional guidelines and menu checklist for residential and nursing homes [44]	—	The Eatwell plate.^c^	—	—	—	Fluid: 1.2 L/day.	—	Fortify drinks, soups, desserts, sauces and vegetables.
								No nutrient targets.
Wales								
Food and Nutrition in Care Homes for Older People [42]	—	Eatwell Guide.	Energy: 7942–10,868 kJ/day (1900–2600 kcal/day).	—	Fibre: 30 g/day.	Fluid: 1.6‐2 L/day.	Omega 3: fish 3 times/week.	Fortify drinks, soups, desserts, sauces, vegetables and mashed potato.
			Protein: 55–90 g/day.				Specific food recommendations to ensure adequate iron, zinc, all B vitamins, folate, vitamin D, calcium, selenium, salt. No amounts specified.	
								Nutrient target: Increase energy by ≥ 2092 kJ (500 kcal) per day.
*United States of America*								
Diet Manual for Long Term Care Resident [43]	—	Dietary Guidelines for Americans and My Plate for Older Adults.	Energy: 6688–8360 kJ/day (1600–2000 cal/day).	—	Fibre: No recommendations for regular diet.	Fluid: ³8 cups or more/day.	No specific recommendations for regular diet.	—
			Protein: 60–75 g/day,					
*Canada*								
Best Practices for Nutrition, Food Service and Dining in Long Term Care Homes, Ontario [20]	—	Based on Canada's Food Guide.	Energy: 125–146 kJ/kg/day (30–35 cal/kg/day).	—	Fibre: 20–30 g/day.	25 to 30 mL fluids/kg.	—	Fortify food, such as milk between meals (if nutritional requirements are not being achieved).
			Protein: 1–1.5 g/kg/day.					
								No nutrient targets.
Menu Planning in Long Term Care [37]	—	Adaption of Canada's Dietary Guidelines to suit LTC.	Energy: 8360 kJ/day (2000 kcal/day).	Fat: 30%–35% of kJ/day.	Fibre: 30 g/day.	Fluid: 2 L/day.	Sodium: 3500 mg or less.	Fortify cereals, soups, appetisers, mashed potato, pasta, desserts and fruit juice.
			Protein: 100 g/day.				Lists all RDI or AI: > 75% RDA/AI daily and 100% average over full menu cycle.	Nutrient targets:
			Based on full menu cycle average.					Hot cereal: 544 kJ (130 kcal) & 10 g per serve
								Soup: 628 kJ (150 kcal) & 8 g per serve
								Mashed potato: 418 kJ (100 kcal) & 3 g per serve
								Dessert: 503 kJ (120 kcal) & 5 g per serve
								Snack: 503 kJ (120 kcal) & 5 g per serve
National Long Term Care Standards Project: Recommended Food & Nutrition Standards (Background Document) [5]	—	—	Energy and protein: > 75% RDA/AI per day and 100% average over menu cycle.	—	Fibre: > 75% RDA/AI per day and 100% average over menu cycle.	Fluid: > 75% RDA/AI per day and 100% average over menu cycle.	Calcium: > 75% RDA/AI per day and 100% average over menu cycle.	Adequate intake is supported by fortified foods. No nutrient targets or fortified food examples provided.
Audits and More: A Nutrition and Food Service Audit Manual for Adult Residential Care Facilities with 25 or more Persons with Care [38]	Recommends calculating a reference person.	Eat Well With Canada's Food Guide.	Energy: 6688–8360 kJ/day (1600–2000 kcal/day).	Carbohydrates: 45%–65% of kJ. Total fat: 20%–35% of kJ, saturated: 10% of kJ,	Fibre: 21 g/day (minimum).	Fluid: 1.5 L/day.	Vitamin C: 122–123 mg/day (minimum) to 2000 mg/day (maximum),	—
			Protein: weekly average within the AMDR of 10%–35% (~equivalent to 40–175 g/day).	Unsaturated fat: 30–45 mL (includes oils, mayonnaise, and salad dressing).			Thiamin: 1.5 mg‐1.8 mg/day (minimum),	
							Riboflavin: 1.4 mg‐1.9 mg/day (minimum),	
							Niacin Equivalent: 20–25 mg/day (minimum),	
							Vitamin B_6_: 1.7 mg–2.1 mg/day (minimum) to 100 mg/day (maximum),	
							Folate:346DFE–408 DFE (minimum) to 1000DFE (maximum),	
							Magnesium: 340–450 mg/day (minimum),	
							Pantothenic acid: 5 mg/day (minimum),	
							Vitamin B_12_: 3.3 μg–4.6 μg/day (minimum),	
							Calcium: 1200 mg/day (minimum) to 2500 μg/day (maximum),	
							Iron: 13mg‐14mg/day (minimum) to 45 mg/day (maximum),	
							Phosphorous: 901 mg–1046 mg/day (minimum) to 3000 mg (maximum)/day.	
							Zinc: 9.8–12mg (minimum) to 40 mg (maximum). Also documented the Upper Tolerable Limits (UL).	

Abbreviations: AGHE = Australian Guide to Healthy Eating, DFE = dietary folate equivalent, NRVs = nutrient reference values.

^a^ Energy has been reported in kJ, standards that reported in calories were converted to kJ.

^b^ Micronutrient requirements for male and female are combined and reported as a range.

^c^ The Eatwell plate includes five food groups including (1) bread rice, potatoes, pasta and other starchy foods; (2) fruit and vegetables, (3) milk and dairy foods; (4) meat, fish, eggs, beans and other non‐dairy sources of protein; and (5) foods and drinks high in fat and/or sugar.

Macronutrient recommendations were made in nine (53%) tools, using either proportion of energy for each macronutrient [32, 35, 37, 38, 41], or weight‐based targets in grams [39, 40], and one tool made recommendations for fat without a specific target amount [34]. Twelve (71%) tools provided recommendations for fibre [5, 20, 31, 32, 33, 34, 35, 37, 38, 39, 40, 41, 42], and 15 tools (88%) stated recommendations relating to fluid [5, 17, 20, 32, 33, 34, 37, 38, 39, 40, 41, 42, 43, 44]. Twelve tools (71%) made one or more specific recommendations for micronutrients [5, 31, 32, 33, 34, 35, 37, 38, 39, 40, 41, 42], with calcium being the most common micronutrient referred to (*n* = 9, 53%). Additionally, 11 (65%) tools made reference to fortifying particular meal items to increase energy and protein content (e.g., milk, porridge) [17, 20, 31, 32, 33, 34, 35, 37, 41, 42, 44] and four (24%) provided specific energy and/or protein targets for fortifying foods [17, 31, 37, 42].

### 3.3 Therapeutic and Special Diets

Recommendations relating to therapeutic and special diets varied across the tools (presented in Table 3). All tools provided guidance on texture modification/fluid consistency, with 11 (65%) referencing the International Dysphagia Diet Standardisation Initiative or a national equivalent texture‐modified/fluid consistency classification system [17, 20, 31, 32, 33, 34, 35, 36, 42, 43, 44]. Four tools made a general reference to texture‐modified/fluid consistency, with no specific reference to a classification system [5, 37, 38, 39] and two tools referred to another document or website that discusses texture‐modified/fluid consistency (e.g., National Association of Care Catering Website [39] or Eating Well for Older People with Dementia [41]).

**Table 3:** Information reported in the menu assessment tools relating to texture modification/fluid consistency, therapeutic and special diets.

Title of document and reference	Texture modification/Fluid consistency, therapeutic and special diets					
	Texture modification/Fluid consistency	Therapeutic modification (nutrient modification only)	Finger food	Cultural and religious recommendations	Vegetarian and vegan	Special occasions and celebrations
*Australia*						
Nutrition Standards for Meals and Menus [31]	Texture classification based on IDDSI; recommends minimum choice for TM meals per day, TM options should follow the regular menu pattern as closely as possible, and to use moulded meals.	Therapeutic modification recommended for (1) nutrient restriction or elimination (e.g., allergies); (2) increasing or reducing nutrients (e.g., low sodium), and quantifying; (3) nutrient amounts (e.g., high protein diet providing > 50 g/day). Recommends integrating therapeutic modified diets into regular menu where possible and dietitian input to ensure nutritional adequacy.	Recommends finger foods to increase choice and variety, provides specifications for choice across the menu cycle.	Recommends accommodating cultural and religious diets within restriction and constraints of food supply issues.	Recommends at least two vegetarian meals, including one vegan meal, should be provided per day, which should contain quality protein sources.	—
Apunipima Remote Residential Aged Care Menu Assessment Toolkit [36]	Texture classification based on Australian requirements.^a^	—	—	Menu planning tool explicitly made for Aboriginal and Torres Strait Islander peoples living in remote communities in Cape York.	Vegetarian meals included on the menu at least 1 time/week. Meals are based on eggs, cheese, tofu, nuts or dried beans.	—
	Recommends RACF to have a texture modified protocol for each texture and fluid type.			No other specific cultural and religious recommendations.	No reference to vegan diets.	
Nutrition and Quality Food Standards for Adults in Victorian Public Hospitals and Residential Aged Care Services [32]	Aligns with IDDSI; recommends nutrient provision and minimum choice aligning with regular texture diet.	Does not provide recommendations or specifications for therapeutic diets, recommends dietitian consultation to support meeting nutritional requirements.	Recommends inclusion of finger foods to promote independence.	Menus planning should meet most the needs of most of the population, including consideration of cultural and religious needs; recommends authentic presentation of cultural and religious meals so they are recognisable and acceptable; recommends including special menu days based on cultural celebrations.	A vegetarian menu choice be available at every eating occasion, using a variety of protein sources including legumes, seeds, nuts, tofu, textured vegetable protein (TVP), milk, cheese and eggs.	Menu choice and variety be further increased when celebrating themed days (e.g., BBQ Day or Football Grand Final Day) and cultural days (e.g., Chinese New Year or Christmas.
Best Practice Food and Nutrition Manual for Aged Care Homes [33]	Refers to IDDSI. The great majority of menu items can be modified for TMD. TM food is presented in a form most convenient to individual residents. A detailed TM menu should be documented and updated as appropriate.	Any therapeutic diet must have clear benefit and not increase risk of malnutrition. A dietitian should be consulted regularly to ensure nutritional adequate of the menu and to ensure that foods provided meet the resident's nutrition needs. Reference to ‘Therapeutic Diet Specifications for Adult Inpatients.’	Food is presented in a form most convenient to individual residents, includes finger foods when needed. A detailed list of finger foods provided.	Cultural, ethnic and religious food preferences and cooking methods are met.	A vegetarian menu must be nutritionally adequate and offer appropriate choices that consider both nutrition and residents acceptability. The type of vegetarian diet required will need to be established, including vegan, lacto‐vegetarian and ovo‐lacto.	On occasions, the menu includes theme or celebration foods. Regular planned activities such as barbeques, happy hour, special occasion and theme meals, including meals from other cultures, not only provide variety to the mealtime, they can stimulate resident interaction.
				A detail section on ideas to maximise food enjoyment for religious, spiritual, cultural and linguistic background of residents.	Vegetarian meals are based on eggs, cheese, tofu, nuts or legumes (e.g., lentils).	
Menu and Nutritional Standards for Public Health Facilities in South Australia [34]	Refers to IDDSI. Specified Nutrition Standards and Minimum Serve Size for TMD. Comprehensive suggestions for TMD provision and delivery to residents.	An appropriately skilled dietitian should be consulted to provide advice on relevant therapeutic diets. Reference to ‘Therapeutic Diet Specifications for Adult Inpatients.’	Finger foods should be available for residents who have trouble with self‐feeding.	Provide adequate variety, quality and quantity for each resident's individual nutrition needs including special dietary, medical, religious or cultural requirements.	No specific recommendations in relation to RACF.	—
		Ensure nutritional adequacy for residents with food allergy through meal choices ethic include substitute or alternative choices, rather than just the omission of the allergen.		A section on considerations for patients from diverse cultural and religious backgrounds.	A section on considerations for nutrition specifications for vegetarian/vegan.	
				Provides links to websites for specific cultural and religious groups.		
Nutrition Standards for Adult Inpatients and Residential Aged Care Policy [35]	Refers to IDDSI. Must still be choice; provide at least one hot main for lunch and dinner and offer a second hot choice option or appropriate frozen meal. Specific recommendations for Level 4 and Level 5. Provides minimum requirement for frequency of TM meal audits.	Specific therapeutic diets (refer to local Food Service Manuals and local dietitian).	—	Menu design is to be based on the needs of the broader population, taking into account social, cultural and religious needs. No specific recommendations.	Vegetarian option at each meal, at least one per day should meet band 1 main vegetarian criteria.	—
					Banding requirements specific to vegetarian hot mains (portion size and energy, protein, fat and sodium requirements per portion).	
*United Kingdom*						
England						
Guidance on Food Served to Older People in Residential Care [40]	No reference to TM diet. Only statement relating to texture‐ consider the texture of the starchy option being provided, you may wish to consider offering an option that is softer to chew when also offering crisper/firmer foods.	Mentions considerations for allergens with each food group. Also references a document ‘National Association of Care Caterers’ for special requirements.	—	Mentions tips for food related customs for each food group.	Meat alternatives for vegetarians should be varied and a variety of foods from the meat/alternative food group should be used. Cheese may be used as a substitute; however, it should not be used too often as the protein source for vegetarians as it can be high in salt and saturated fat. Beans and pulses – for example, baked beans, butter beans, kidney beans and lentils are in this group and provide a good source of protein for vegetarians and are low in fat. Other textured protein products suitable for vegetarians, such as tofu, soya, quinoa and quorn (R).	—
Eating Well for Older People [41]	Refers to Eating Well for Older People with Dementia for guidance on TM diet.	Nutrition information on how to manage malnutrition, anaemia, digestive disorders, diabetes mellitus, muscle and bone disorders, mouth problems, swallowing difficulties, overweight and coronary heart disease and stroke.	—	Every effort should be made to make the eating environment as attractive and as culturally appropriate as possible.	Many older people enjoy meat dishes, but future generations will need a much broader menu, including vegetarian meals.	—
				Providing variety and choice‐ exploring likes and dislikes is particularly important where there may be ethnic, religious and cultural requirements.		
				Checklist to assess the quality of food and equipment for cultural and religious requirements.	Examples of meals suitable for different cultural backgrounds which includes vegetarian meals.	
Healthier and More Sustainable Catering. A Toolkit for Serving Food to Older People in Residential Care [39]	Refers to Dysphagia Diet Food Texture Descriptors on the National Association of Care Catering Website.	Noted to be aware of food sensitivities (allergies, intolerances and coeliac disease).	—	Caters need to prepare for cultural and religious food sensitives and also be aware that some individuals/groups may fast on occasion. Fasting may involve exclusion of meat and/or other foods at specific times.	Meat and fish alternatives for vegetarians and vegans should be varied and a variety of foods from this group should be used. Cheese may be used as a substitute for vegetarians. However, it should not be used too often as a source of protein as it can be high in salt and saturated fat.	—
Care Home Digest, Menu planning and food service guidelines for older adults living in care homes [17]	Refers to IDDSI. Provides information on IDDSI testing methods and mealtimes, mealtime planning, hints and tips for food preparation, choice, and hydration recommendations.	List diets for therapeutic or medically required to treat/manage a medical condition. Mentions specific diets and/or medical conditions such as food‐based nutrition support for residents who are at risk of malnutrition, dysphagia, dementia, diabetes, healthier eating for residents who wish to lose weight, mental health conditions, palliative/end of life care, food allergy and kidney (renal) disease.	Ensure that a finger food meal or snack is comparable to the regular menu options—finger food options should be as nutritious as other regular meals and snacks. Provides tips for providing finger food options such as present finger foods in an accessible way, provide a variety of hot and cold options and residents with swallowing difficulties or requiring special diets will need further consideration. An extensive list of finger food examples at different mealtimes is documented.	Provides a detailed list of different religions and also examples of cultural considerations.	It is essential that vegetarian and vegan residents’ dietary needs and preferences are considered when menu planning.	Social occasions such as specific events can keep options interesting and exciting for all residents in the home.
		If the catering team is not familiar with the therapeutic, they should discuss the specific needs with the resident, their family and/or friends where appropriate and the medical team that has recommended the therapeutic diet.		Seven ideas for menu planning are described such as knowledge and skills of catering team, working with the resident and their family, standard menu should be adapt or if 50% of residents within the home have similar religious or cultural preferences, the standard menu should include at least one appropriate option per day, offer variety, consider snack times and consider how the menu is presented.	Where appropriate, including a variety of vegetarian or vegan options as part of the regular menu can provide more choice for all residents. A vegetarian or vegan diet should meet a resident's nutritional needs and including soya, tofu, calcium fortified soya milk, fortified breakfast cereals, nuts and beans can help to support residents to eat a nutritionally balanced diet. However, additional consideration may be required to ensure that nutritional balance can be achieved, as some vegetarian and vegan foods may be lower in protein and micronutrients, including vitamin B_12_, folate, calcium, iron, zinc and iodine. Detail list of energy and protein content of some vegetarian and vegan foods and meal suggestions are provided.	
					Where there are longer term challenges with meeting residents’ nutritional needs, professional advice from a dietitian is recommended.	
Northern Ireland						
Nutritional guidelines and menu checklist for residential and nursing homes [44]	Refers to National Dysphagia Food Texture Descriptors. All therapeutic diets should be given adequate choice and variety, no specific targets provided.	All therapeutic diets should be given adequate choice and variety, no specific targets provided.	Encourages finger foods for residents with dementia. Provides examples of finger foods and meals.	Staff should ensure food is acceptable and in keeping with ethnic, cultural and religious requirements of individuals. Additional choices should be available to all religious and cultural groups. No specific targets.	Provides definition of a vegetarian or vegan diet and one example of a vegetarian dish in a menu. No other recommendations.	—
Wales						
Food and Nutrition in Care Homes for Older People [42]	Refers to IDDSI. If a health professional has accessed a resident and requires a specific texture modification from one of the levels described by IDDSI this needs to be followed.	Provision for special diets for medical reasons (diabetes, coeliac disease, allergies, weight management).	Finger foods can provide a nutritious and enjoyable alternative to plated meals. They can also be used to provide extra nutritious snacks. Provides a summary of who may benefit from finger foods and important considerations when offering finger foods.	It is essential for care homes to recognise, respect and cater for different religious, philosophical and cultural needs. Dietary practices between and within different faiths can be diverse, and it is important not to make assumptions. A summary of different cultural, philosophical and religious consideration and different food products is listed in a table.	Provides definition of a vegan diet and that vegan diets may be low in a number of nutrients.	Using special occasions or themed meal times can also help to add variety and interest.
	Whole section on Eating and drink well using TM diet and IDDSI.			Specifies: (1) care homes can utilise the range of cultural aspects in theme days and events to share periods of celebration and social activities. (2) Some cultures have periods of fasting. Must be respected by the care home. (3) Specific dietary requirements are different to likes and dislikes.	Provides an overview of important considerations for vegetarian and vegan diets and links to websites with more information on vegan diets.	
	Meal examples for Level 6 and Level 4.					
*United States of America*						
Diet Manual for Long Term Care Residents [43]	Detailed description, approximate composition, adequacy and meal plan for the following diets: Mechanical Diet (Dental Diet), Dysphagia Level 1 (Pureed Diet), Dysphagia Level 2 (Mechanically Altered Diet), Advanced Level 3 Diet.	Detailed description, approximate composition, adequacy and meal plan for the following diets: full liquid diet, clear liquid diet, restricted fibre diet/restricted residue diet, increased fibre diet, pleasure feedings, no added salt diet, low sodium diet (2–4 g), cholesterol restricted and fat controlled diet, limited K+ diet and liberalised renal diet, renal diet, high phosphorus foods, carbohydrate controlled diet, calorie restricted (low calore) diet, limited concentrated sweets (LCS) diet, diabetic diet, lactose reduced diet and gluten free diet.	Finger Food Diet‐ a regular diet consistency diet which can easily eaten with dingers and not requiring silverware. Policy to offered to resident identified as having difficulty efficiency feeding themselves with utensils, possibly leading to risk of poor nutrition.	Food choices satisfy resident cultural preferences.	Detailed description, approximate composition, adequacy and meal plan for the following diets: vegetarian diet, lacto‐ovovegetarian meal plan and vegan meal plan.	—
				Detailed description, approximate composition, adequacy and meal plan for Kosher Diet.		
*Canada*						
Best Practices for Nutrition, Food Service and Dining in Long Term Care Homes [20]	Refers to IDDSI. Provides information on considerations for LTC homes to consider in implementing IDDSI‐ including initial plan, food preparation and communications/training.	Therapeutic diets are created using the regular menu as a base (providing similar choice, variety and palatability) and used as needed, based on dietitian and interdisciplinary care team's assessment. Therapeutic diets are sufficiently flexible to allow for liberalisation where appropriate.	Finger foods can be easily picked up and eaten with hands. Residents with dementia may benefit from finger foods or for residents who wander or be impatient).	Menus reflect current residents’ social, ethnic, cultural and religious practices and needs.	When several residents follow a similar diet (e.g., vegetarian) then a standard menu may be considered.	A residents’ food committee can be established for planning and approving special occasion menus (e.g., holiday meals, special functions, barbecues).
	Texture modified menus follow the regular menu as closely as possible to provide similar choice, variety and palatability.		Finger foods may be considered a type of texture modification.	There is a policy and procedure that addresses the needs of residents who request cultural or religious‐specific food choices.		
Menu Planning in Long Term Care [37]	Modified textured menus follow the main menu as closely as possible and provide equivalent nutrients.	Specialised advice from a dietitian for those with health conditions and/or in a clinical setting.	Fingers foods promote adequate intake or those at risk of malnutrition and with swallowing and/or chewing difficulties.	The culture/ethnicities of the resident population are considered during menu planning and appropriate cultural foods are included on the menu.	For those who consume a vegetarian diet (especially vegan) is important to offer a variety of different plant‐based protein sources to ensure that all essential amino acids are consumed in adequate amounts.	—
National Long Term Care Standards Project: Recommended Food & Nutrition Standards [5]	Texture modified menus follow the regular menu as closely as possible to provide similar choice, variety and palatability. The TM diet can be demonstrated to provide > 75% of the Recommended Dietary Allowance (RDA)/Adequate Intake (AI) on any specific day, while achieving an average of 100% of RDA/AI over the duration of the menu for energy, protein, calcium, dietary fibre and fluid.	Therapeutic modified menus follow the regular menu as closely as possible to provide similar choice, variety and palatability.	Use of finger foods (as indicated in an individual's nutrition care plan).	Menus reflect current residents’ social, ethnic, cultural and religious practices and needs.	Mentioned vegetarian and vegan diets, however, did not make any specific recommendations.	—
	Staff involved in meal and snack service receive education/training on nutrition, texture modification and hydration.			There is a policy and procedure that addresses the needs of residents who request cultural or religious‐specific food choices.		
	Discusses IDDSI, however no specific recommendation.					
Audits and More: A Nutrition and Food Service Audit Manual for Adult Residential Care Facilities with 25 or more Persons with Care [38]	References TM but does not describe any classification system. Recommends TM menus follow the main menu as closely as possible to provide the same choice.	Recommends therapeutic modified menus follow the main menu as closely as possible to provide the same choice.	—	Persons in care should be interviewed to obtain information on specific cultural and religious food practices to be incorporated into the menu.	Food provided should meet personal preferences and requirements, including vegetarian diets. Vegetarian meals should be offered on a regular basis but does not define how often is considered regular.	Menus provide regular opportunities for meals chosen by those in care (e.g., on their birthday) and special events (e.g., Robbie Burns Day). Special occasions, holidays and birthdays are celebrated and catering for special events is considered.

Abbreviations: IDDSI = International Dysphagia Diet Standardisation Initiative, TM = Texture Modified.

^a^ Pre‐IDDSI.

Sixteen tools (94%) discussed therapeutic diets, of which only four tools made specific recommendations for therapeutic diets and listed specific diets (i.e. diets for weight management or gluten‐free diets for coeliac disease) [17, 31, 41, 43]. Six tools (35%) highlighted the need for dietitian involvement for residents requiring therapeutic diets [20, 32, 33, 34, 35, 37], and four tools (24%) made reference to other resources with details on therapeutic diets [33, 34, 35, 40]. Five tools (29%) highlighted the importance of having a therapeutic menu that follows the regular menu [5, 20, 31, 33, 38]. Finger foods were included as a recommendation in 11 tools (65%) [5, 17, 20, 31, 32, 34, 35, 37, 42, 43, 44]. All tools made reference to the importance of ensuring the menu meets the cultural and religious needs of residents [5, 17, 20, 31, 32, 33, 34, 35, 37, 38, 39, 40, 41, 42, 43, 44]. All tools made reference to vegetarian/vegan meals, with seven (41%) specifying vegetarian/vegan menu or every day offering requirements [17, 20, 31, 32, 33, 35, 43], four (24%) mention vegetarian/vegan but did not provide specific recommendations [34, 37, 42, 44], three (18%) provided meat alternative recommendations [39, 40, 41], one (6%) mentioned providing vegetarian meals (did not specify vegan) once per week [36] and the last tool (*n* = 1, 6%) stated offering vegetarian/vegan meals on a ‘regular basis.’ [38] Six tools (35%) discussed facilitating and considering menu changes for celebrations or special occasions [17, 20, 32, 33, 38, 42].

### 3.4 Foodservice Management Recommendations

The recommendations provided in the tools relating to foodservice management are provided in Table 4. Twelve tools (71%) provided recommendations for frequency of completing menu reviews, which varied from every 3 months [41], every 6 months [17, 32, 36, 38, 42], annually [5], every 2 years [31, 34], seasonally [37], with any changes to the menu [5, 17, 31, 34], and two stating reviews were recommended, however provided no time frame [39, 44]. Twelve tools (71%) discussed cost and budgeting considerations [5, 17, 20, 31, 32, 34, 36, 38, 39, 41, 42]. Three tools (18%) provided a recommendation that ‘budgets should be used’ [31, 32, 38], whereas another three tools provided recommendations for cost per food group [36] or cost per resident [20, 41]. Two tools specified that cost should not be at the expense of quality food ingredients [34, 41], one referred to specific buying standards [39] and three tools provided strategies on how to reduce cost, for example, bulk buying [42] or using ready prepared meals to manage limited labour [17, 37]. Over half (*n* = 9, 53%) of the tools provided information relating to food safety, with six of those tools specifying compliance to a Food Standard or Food Act [32, 34, 35, 38, 41, 42], and two tools provided food safety tips for food groups [39, 40].

**Table 4:** Information reported in the menu assessment tools relating to foodservice management recommendations.

Title of document, organisation/author, year of publication	Frequency of menu review	Budget/Cost recommendations	Food safety	Dining experience/Environment	Menu availability, presentation and details for residents	Sample menu or recipes	Software analysis	Staff training	Sustainability considerations
*Australia*									
Nutrition Standards for Meals and Menus [31]	Every 2 years or with significant menu changes.	Recommends that budget should be used to guide your menu development.	—	Recommends that food and dining environment should meet the nutritional and psychosocial needs of all residents.	—	Sample menu provided; sample recipes not provided.	—	—	—
Apunipima Remote Residential Aged Care Menu Assessment Toolkit [36]	Every 6 months	Provides guidance that services may use to plan for future budget.	—	—	—	Sample menu provided; sample recipes not provided.	FoodWorks recommended.	—	—
		Menu planning principles such as budget should be used to guide your menu development.							
		Recommendation for % of total cost expenditure: 50% fruit, vegetables, breads and cereals, 40% meat and milk, 10% other foods.							
Nutrition and Quality Food Standards for Adults in Victorian Public Hospitals and Residential Aged Care Services [32]	Every 6 months	Recommends that organisational management is responsible for budget. Evaluate budget constraints and identify potential additions or modifications.	Provides specific considerations for vulnerable populations as per Australia New Zealand Food Standards Code.	Strategies to enhance the meal environment provided, including improved ambiance, decor and physical environment, avoiding distractions, improving meal delivery, seating options, menus and quality audit tools provided.	Menu items have commonly accepted and understood names and/or a description that accurately reflects the contents of the dish for ease of patient/resident/family recognition.	Sample menu and recipes provided.	Recommends Foodworks software.	Regular training for food safety in alignment with FSANZ, the IDDSI framework, and the National Allergy Strategy to be taken and documented for all staff involved in producing and delivering meals.	Section on sustainability and procurement. Specific recommendation: (1) health services minimise the number of packets on a meal tray—for example, decanting cereals into a bowl and juice into a glass from bulk sources, (2) food waste management plans integrate with the Victorian Government's Sustainability in Healthcare Environmental sustainability strategy 2018–19 to 2022–23, (3) Health services ensure that, where possible, foods are seasonal and sourced from local or Victorian producers, and (4) health services consider developing an organisational local food procurement policy.
					Pictorial and translated menus are available where there is an identified need in the health service population assessment.				
Best Practice Food and Nutrition Manual for Aged Care Homes [33]	—	—	—	Recommends consideration of ambiance and environment enhancements through use of colour, lighting, air temperature, background music, avoid distractions during meals, furniture, cooking smells, table settings and appointments, routinely providing 3 main and 3 mid meals, emphasise catering to individual needs, and creating a relaxed not rushed environment.	The written menu has details of all food and beverages offered at both main meals and mid‐meals, that is, the type of soup, the actual vegetables, and the range of beverages.	Sample menu and recipes provided.	—	—	—
Menu and Nutritional Standards for Public Health Facilities in South Australia [34]	Every 2 years or with significant menu changes.	Recommends that cost control should not be at the expense of quality food ingredients.	States that Public Health Facilities have a legal responsibility to ensure safety and suitability of food supplied to residents, and must comply with Australia Food Act and the Australia New Zealand Food Standards Code.	Recommends providing a home‐like environment in the dining room.	To ensure all consumers can participate in menu selection and have an understanding of meals being provided, appropriate menu and food description translation resources (written and/or verbal) should be available.	Sample menu provided; sample recipes not provided.	—	—	—
Nutrition Standards for Adult Inpatients and Residential Aged Care Policy [35]	—	—	Recommends following National Safety and Quality Health Service Standards.	—	—	Sample recipes provided; sample menu not provided.	Recommends Foodworks software.	—	—
*United Kingdom*									
England									
Guidance on Food Served to Older People in Residential Care [40]	—	—	Tips are provided based on food group (starchy, fruit/vegetables, milk and dairy, meat and meat alternatives, foods and/or drinks high in fat and sugar).	—		Sample menu provided; sample recipes not provided.	Recommends using software with up‐to‐date information, no specific recommendation.	—	—
Eating Well for Older People [41]	Every 3 months	Recommends that cost should not override the need for adequate nutritional content in planning and preparation of food.	References publications from Food Standards Agency in terms of suppliers.	Provides dining environment strategies for ‘exciting the appetite’, including the presentation of tray meals, the way the food looks on the plate, the attitude of the staff, and creating social occasions.	The registered person ensures that there is a menu (changed regularly) offering a choice of meals in written or other formats to suit the capacities of all service users, which is given, read or explained to service users.	Sample menu and recipes provided.	Recommends CORA Menu Planner program. The program contains a recipe library database of over 800 dishes, snacks and drinks, complete with a nutritional analysis of each item and recipes where appropriate.	—	—
		Guide: Stipulates that at least 18 pounds (2004 prices) be spent per resident per week should be spent on food ingredients to ensure that food of sufficient nutritional content can be made available.							
Healthier and More Sustainable Catering. A Toolkit for Serving Food to Older People in Residential Care [39]	Reviews recommended; no specific timeframe provided	References the Government Buying Standards for Food and Catering Services; however, cost is only a consideration.	Tips are provided based on food group (potatoes, bread, rice, pasta and other starchy carbohydrates, fruits and vegetables, dairy and alternatives, beans, pulses, fish, eggs, meat and other proteins, oils and spreads, and foods high in fat, salt, sugars.	—	—	—	—	—	Large sustainability focus. The information presented in this toolkit supports Government Buying Standards for Food and Catering Services (GBSF) to enable the production of food to higher sustainability and nutritional standards, and more sustainable catering service provision.
Care Home Digest, Menu planning and food service guidelines for older adults living in care homes [17]	Seasonally, at least 6 monthly or more frequently if feedback requires it to be adjusted sooner.	Care homes have to be mindful of the need to design menus that are value for money. In line with homes contractual requirements, budgets must be sufficient to buy both enough food to meet residents’ needs and to provide appropriate equipment for its safe production and service. Effectively designed menus which plan for the use of seasonal produce and minimise food waste ensures the efficient use of a food budget. It is important to know how much a menu will cost and to agree the budget and period covered (daily/weekly/monthly) from the start of any menu planning cycle. It is expected that the budget for food will be increased at each review to ensure that inflationary increases in food costs are planned for.	Lists food hygiene and safety requirements as a factor that may influence which dishes can be included in a menu.	Provides guidelines and team considerations for the dining room environment, preparation for meal service, during mealtimes, after mealtimes and room service delivery. Also includes a checklist tool for homes to use to observe and measure their meal service can be found.	Daily menus should be displayed at an appropriate height for residents to be able to read them, in a font size and typeface that makes them easy to read. Table menus stating the meal choices at each mealtime should be available and should also use a suitable font size and typeface. Pictorial menus should also be available for those who might need different support to be able to choose their meal. Menus can be used with residents before the meal service to talk about the next meal as this might support them to be tempted to eat.	Sample receipes provided.	—	Training about the new menu should be provided to food service teams and carers, covering key updates on the menu and any operational changes. Catering and care teams receive training about the management of food allergies and how to safely provide food for anyone with a food allergy	When considering sustainability, thought needs to be given to how food is grown, bought, stored, cooked and wasted. Ideas that could be considered include: (1) use meat, dairy products and eggs that are produced within high animal welfare standards and fish that is from a sustainable source, (2) recycle packaging, (3) work with suppliers to determine the sustainability of the food being ordered, (4) the number of ingredients and the cooking methods used, (5) purchase seasonal foods with a shelf life that allows it to be used up before going out of date and (6) minimise food waste and use left over food where possible and safe to do so. Refers to Delivering a ‘Net Zero’ NHS
Northern Ireland									
Nutritional guidelines and menu checklist for residential and nursing homes [44]	Reviews recommended regularly, does not provide timeframes.	—	—	—	Menus should be clearly written in familiar language and displayed in a suitable format and location so that residents and their representatives are aware of what is available at each mealtime.	Example menu provided.	—	Recommends all staff should receive training on how to manage a choking incident, and dysphagia/swallow awareness training. Nutrition training to staff and carers encouraged.	—
Wales									
Food and Nutrition in Care Homes for Older People [42]	Twice per year.	States that sufficient menu planning can reduce overall cost. Recommends that buying tinned/frozen foods in bulk can reduce costs.	Provides overview of food hygiene and safety practices covered by regulations and refers to appropriate guidance and legislations.	Recommends developing a positive dining environment and experience to encourage residents to eat well.	Menus include details of all meal choices, snacks and drinks that are offered.	Example menus provided.	—	Recommends Food Hygiene training for all care staff, with food handlers completing a Level 2 Food Hygiene course (within 3 months of starting work).	
								Kitchen managers, chefs and cooks undertake Level 3 Food Hygiene training (updated at least every 3 years).	
Advised that kitchen staff and food handlers have training on food allergens.	Menu planning can be used to reduce the use of those ingredients with a high environmental impact and will also reduce food waste.								
	Provides sustainability recommendations, including (1) use local and in‐season ingredients when possible, (2) ensure meat, dairy products and eggs are produced within high animal welfare standards, (3) look out for the following quality assurance standard logo's, (4) avoid red list or endangered species of farmed or wild fish, (5) use local suppliers where possible and try to cook as much of the food on site.								
					Menus are in a user friendly format for all resident's needs (e.g., large print, visual) and are displayed.				
*United States of America*									
Diet Manual for Long Term Care Residents [43]	—	—	—	Provides link to ‘The Culture Change Movement. New Dining Practice Standards.’	—	Example menus and recipes provided,	—	—	—
*Canada*									
Best Practices for Nutrition, Food Service and Dining in Long Term Care Homes [20]	—	Recommendations based on surveys: 1.1 Minimum required spending/budget for resident meals is set at provincial/territorial level and adjusted annually for changes in the Consumer Price Index across regions.	—	States that a pleasant dining environment positively affects quality of life and intake; the physical aspects of the dining location and individualised person‐centred care are determinants of the pleasantness of the dining environment; staff and volunteer resources are main limitation to providing feeding assistance; and availability of feedings should be a high priority to promote independence.	Weekly and daily menus are posted in a common area in or near the dining room for residents and families to see. Font size is as large as is possible and practical.	—	Recommends using appropriate software, no specific recommendations.	All home staff receive orientation to food and nutrition services upon hire. Staff involved in meal and snack service receive education/training on nutrition and hydration. Topics may include: (1) basic therapeutic diets, (2) food texture, (3) fluid consistency, (4) food safety, (5) customer service/hospitality training, (6) knowledge of dementia and responsive behaviours, (7) ability to recognise, report and document signs and symptoms of dysphagia eating assistance, (8) person/relationship‐centred care and (9) promoting and improving the mealtime experience.	—
		1.2 The food budget is dedicated and protected from being spent on other factors.							
		1.3 Tube feeding and supplement budgets are established at provincial/territorial level and separated from the food budget.						All Food/Nutrition Staff receive education/training on topics such as: (1) food safety, temperature control, dining service, nutrition related health concerns and other topics as needed, (2) proper preparation, testing and storage of all levels of texture modified foods and thickened fluids to ensure production of food and fluids consistent with developed texture expectations.	
		1.4 Menu changes based on financial analysis and meeting budget goals are reviewed and approved by a Registered Dietitian or Member of Canadian Society of Nutrition Management (CSNM).							
Menu Planning in Long Term Care [37]	Seasonally	Provide specified funding for a raw food allocation, or a minimum food expenditure per resident, to support a healthful menu. Provide sufficient funds towards skilled labour allocation to decrease reliance on highly processed or prepared foods of poor nutritional quality and to promote more healthful foods made in‐house tailored to residents’ needs and preferences.	—	—	—	Sample menu provided; sample recipes not provided.	Recommends using nutrition analysis software, no specific recommendations.	—	—
National Long Term Care Standards Project: Recommended Food & Nutrition Standards [5]	Annually, or when the menu is updated	Minimum required spending/budget for resident meals is set at provincial/territorial level and adjusted annually for changes in the Consumer Price Index across regions.	—	Dining service practices in the LTC home under the supervision of a Registered Dietitian or member of Canadian Society of Nutrition Management. Food intake and quality of life of residents is supported by a pleasant dining environment.	The menu is planned and posted at least a week in advance in a manner accessible for viewing by residents, staff, and visitors.	—	Recommends using a software, however, acknowledges that there is a cost and should be considered a basic requirement for practice in the LTC setting.	Recognises the importance of staff training, however, has no specific recommendations.	—
		The food budget is dedicated and protected from being spent on other factors.							
		Menu changes based on financial analysis and meeting budget goals are reviewed and approved by a Registered Dietitian or member of Canadian Society of Nutrition Management (CSNM).							
Audits and More: A Nutrition and Food Service Audit Manual for Adult Residential Care Facilities with 25 or more Persons with Care [38]	Twice annually	Not recommendations provided; however, states cost should be a consideration in menu planning.	Recommends contacting the regional environmental health office to obtain food safety standards for residential aged care.	Recommends that staff should provide assistance to prepare them for the dining experience, provide a safe, relaxed, supportive and restorative dining environment, provide choice and social opportunities, make provisions for those who do not attend a meal of snack time, provide organised dining and sequence of service, recognise the right to respect, dignity and privacy of persons in care with practices around treatments at mealtime, involve persons in care in food service. Provides a dining environment audit which is recommended to be completed annually.	The menu is posted in the dining area.	Sample menu provided; sample recipes not provided.	Recommends using software for nutrient analysis; no specific recommendations provided.	—	Sustainability in Food Services Checklist, with elements relevant to menu planning, including: (1) vegetarian meals offered on a regular basis, (2) bulk condiments are used (individual portions only used as needed) and (3) reduce food waste by use of forecasting, standardised recipes and plate waste audits.

Over half of the tools (*n* = 10, 59%) provided information or recommendations relating to mealtime experience or eating environment [5, 17, 20, 31, 32, 33, 34, 38, 41, 42], with one tool referencing a standard, ‘The Culture Change Movement New Dining Practice Standards’. [43] Five tools (29%) specified the importance of meeting residents’ needs in relation to their mealtime experiences [17, 20, 31, 34, 42], and five tools (29%) provided strategies for improvement [17, 32, 33, 38, 41]. Ensuring the menu is available and presented (i.e., in the dining room) to residents was recommended by eight tools [5, 17, 20, 32, 33, 38, 41, 44]. Twelve tools (71%) provided sample menus [31, 32, 33, 34, 35, 36, 37, 38, 39, 40, 41, 42, 43] and five tools (29%) shared example recipes [17, 32, 33, 41, 43]. Nine tools (53%) recommended using software for nutritional analysis [5, 20, 37, 38, 40], with four of those tools providing a specific software recommendation [32, 35, 36, 41]. Five tools (29%) recommended staff training in food and nutrition, with specific mentions of food safety/food hygiene, swallowing and/or allergens [17, 20, 32, 42, 44]. Five tools (29%) reported sustainability considerations for menu assessments [17, 32, 38, 39, 42].

## 4 Menu Assessment Guideline Quality

All tools were evaluated using the AGREE II Instrument, with scaled domain scores and overall quality scores listed in Table S1. For scope and purpose (Domain 1), the majority of tools (*n* = 15, 88%) identified the overall objective of the tool, the health questions answered and the population to which the tool applied to [5, 17, 31, 32, 33, 34, 35, 36, 37, 38, 39, 40, 41, 42, 44]. Two tools did not receive a score of ≥ 50% for Domain 1 [20, 43]. In stakeholder involvement (Domain 2), almost half (*n* = 8, 47%) of the tools involved stakeholders in their development [5, 31, 32, 33, 34, 38, 41, 44]. No tool received a score of > 50% for rigour of development (Domain 3). Tools commonly did not describe the process for searching or selecting and synthesising evidence to form the recommendations. Additionally, not all guidelines were reviewed by external experts before publication. Most tools (*n* = 13, 76%) provided a clear presentation of information (Domain 4), with specific and easily identifiable recommendations [5, 17, 31, 32, 33, 34, 35, 36, 38, 41, 42, 44]. For applicability of the tools (Domain 5), seven (41%) tools scored < 50% as they did not describe the facilitators and barriers or report the consideration of resource implications of the recommendations [5, 34, 35, 39, 40, 43, 44]. Lastly, only one tool scored > 50% for editorial independence (Domain 6) [17]. The majority of the tools did not report the source of funding to develop the guidelines, or the process used for managing/recording conflicts of interest. The overall quality of the tools ranged from two to six (out of a possible seven). Six tools (35%) scored > 50% in four or more domains on the AGREE II and scored four or more for overall quality and were therefore recommended for use with modifications [17, 31, 32, 33, 38, 41]. The remaining eleven tools (65%) were not recommended for use [5, 20, 34, 35, 36, 37, 39, 40, 42, 43, 44] and no tools were recommended for use (without modifications).

## 5 Discussion

This study identified and synthesised existing literature regarding the content areas, recommendations and quality of the publicly available tools to conduct menu assessments for RAC. Overall, there was high variability in the content covered and the recommendations made within the content areas. The overall quality of the tools varied, and no tools meet the quality criteria to be recommended for use in their current form. Tools lacked rigour in their development and/or did not use appropriate or any evidence to underpin the recommendations suggested. The lack of consistency in content areas, recommendations and quality of MATs indicates the need to conduct research to develop international consensus on the detail and development of MATs. Additionally, with no tools being recommended for use, there is a need to modify existing tools to improve their quality or development of a new evidenced‐based MAT to meet current or upcoming menu assessments legislative requirements in RAC [10, 20, 21].

The tools included in this review differed considerably in the breadth of content areas and the specificity of recommendations for menu assessment. No tool covered all content areas, and many did not provide clear guidance or criteria to assess components of the menu assessment. For example, whilst many tools recommended the importance of providing choice or variety, only six tools specified the amount of choice that should be provided for food groups across meals, and four tools provided specific criteria to assess the variety of options in menus. The lack of specific recommendations relating to choice is consistent with the findings of a 2022 published scoping review, which noted the ambiguity around the term choice and the lack of clear guidance for providing choice to residents in RAC [29]. Additionally, the lack of prescriptive guidance was also identified in many tools regarding resident input into the menu. While tools highlighted the importance of resident input, very few provided details for how this feedback should be obtained and incorporated into menus. Without such specific detail, it requires individual dietitian's judgement, which may not reflect best practice and could lead to variability in conducting menu assessments. Lack of prescriptive guidance may be problematic for new graduate dietitians who may be less confident in some knowledge and skill areas [45]. It is, therefore, not surprising that dietitians have emphasised the need for food and nutrition guidelines in RAC and a comprehensive national MAT to support consistency across the sector [46].

The tools included in this review lacked reference to original research with many tools being based on expert opinion and no tools scoring > 50% for rigour of development. Lack of research underpinning MATs was also noted in a recent scoping review [22]. Within this review, lack of original research was particularly apparent within the nutritional targets of the menus assessments. For example, tools that used energy and protein requirements, all had used different reference persons in terms of age, sex height and weight for calculating energy and protein requirements resulting in a wide range of the nutritional targets. Whilst there are recommendations [47] and guidelines that exist at the individual older person level, for example, ESPEN guidelines suggesting a minimum requirement of protein (1 g per kg of body weight per day) and ‘*the amount should be individually adjusted with regard to nutritional status, physical activity level, disease status, and tolerance’* [48], no research to date has provided specific evidence to support appropriate amount of nutrients a menu should provide. In fact, a recent Food and Nutrition Guideline emphasised the importance of determining an appropriate subset of nutrients that could be used as a proxy for the nutritional adequacy of a menu [5]. Additionally, for tools that used food groups, most were based on national dietary guidelines developed for the general, healthy population, for example, some Australian based tools used the Australian Guide to Healthy Eating (AGHE) [49]. The focus of the tools on general population guidelines is particularly concerning given the AGHE (or country equivalent) is not considered to be suitable for the RAC population group [10], as residents typically have different nutrition requirements or face challenges in meeting their nutrition needs [8, 13, 48, 50]. The use of different reference persons and population‐based dietary guidelines may be due to the lack of evidence in the literature specifying the appropriate nutritional targets for RAC menus, which has led to the reliance on expert opinion [5]. Given this, the review supports recent calls for more research to be conducted in RAC [51], with a focus on evidence to underpin a MAT.

The tools included in this review were broad in type and design, with different document types included. The variation of documents may be a contributing factor to the variability of content areas covered and the specificity of recommendations. No tool comprehensively covered all content areas, and as such, dietitians may need to use multiple tools to conduct a menu assessment. Whilst the tools were often focused on menu assessments, many also included other aspects of foodservice management (e.g., dining environment), that may impact nutritional intake and QoL for residents. This review highlights the diverse nature of tools that include a menu assessment component but also raises broader discussion around developing consistency for the assessments required to evaluate RACs foodservice and nutrition practices. For example, best practice guidelines for nutrition, foodservice and dining in Canadian long‐term care homes highlight that organisation, administration (including staffing and budget), menu planning and analysis, food production, nutrition and hydration care and meal/mealtime experience are key areas to be assessed to support enhancement of foodservice and dining experiences [5, 20]. The need for guidelines related to foodservice and nutrition practices is particularly important within the Australian context given there are no nationally recognised food and nutrition guidelines or tools for RACs, which support the implementation of the new Australian Quality Standards [21]. Future research is need to identify and synthesise mealtime assessment tools available within the literature, to identify which tool could be included in the Menu and Mealtime Assessment Tool [21].

Dietitians or a nutrition team developed a number of tools included in this review, with only some (*n* = 8, 47%) tools involving stakeholders [5, 31, 32, 33, 34, 38, 41, 44]. Whilst dietitians are important in the development process as they are the qualified nutrition professional who will likely complete the menu assessment, it is imperative to have other stakeholder input. In Australia, it is common for dietitians to not attend RACs regularly [52]; therefore, other stakeholders, such as chefs, foodservice, care or nursing staff, may need to refer to the MAT or action the recommendations. Moreover, other disciplines may provide other perspectives for designing a MAT, which may be missed by dietetics. Most importantly, input of the resident is essential to ensure a menu is developed that meets residents’ personal preferences, nutritional and cultural needs. Resident input is particularly important, as residents may have different priorities and preferences regarding their nutrition and the systems that support food delivery compared to dietitians or other RAC staff [13, 53, 54, 55]. As such, this highlights the importance of a MAT that is developed in consultation with all relevant stakeholders that are involved in food provision in RAC, including chefs, foodservice, care or nursing staff, other allied health professionals and also residents to ensure menus are designed with residents’ priorities and staff capability in mind.

Quality assessment of the included tools varied in quality, with no tool recommended for use in their current form. Six of the 17 tools recommended for use but with modifications. No tools received a score of > 50% for rigour of development and only one tool scored for editorial independence. The six tools recommended for use with modifications should be revised to include information and detail (either to the current tool iteration or version of the tool), focusing on the domains that scored below 50% in that tool. Additionally, to improve the quality of future tools, tools should specify the methodological process used to develop the tool, including searching, selecting and synthesising evidence to inform recommendations. Future tools need to also incorporate and specify external experts’ reviews prior to publication, report any funding used and declare/manage conflicts of interest. These focus areas are imperative to support transparency and rigour of tool development. Moreover, with tools varying in quality and no tools being recommended for use without modification, it is not surprising that anecdotally, Australian dietitians have reported developing their own MATs. The variation in tool quality and design highlights the need for a comprehensive high‐quality MAT, which dietitians have recently called for [46]. Additionally, the AGREE II Instrument could be used as a framework for future development of MATs to ensure methodological rigour and transparency of tool development.

This review provides first insights into the content and quality of MATs for RAC and provides recommendations for future research and development of a MAT. Moreover, the quality of the review was maximised at all stages by using systematic and rigorous processes (to reduce bias and complexities associated with the synthesis of findings); however, some limitations exist. The inclusion of English‐only articles and only including publicly available tools may have limited the inclusion of all MATs available globally. Additionally, the included content areas of the MAT were decided based on the consensus of the authors (all dietitian‐researchers), as such, it is possible RAC staff or residents may consider other content areas to be important. Lastly, while AGREE II provided a structured approach to review the quality of tools, the AGREE II lacks guidance on how to report and interpret the scores to determine overall quality and recommendations for use [30, 56]. As such, this review supports previous recommendations [30, 56] for the AGREE II to provide greater interpretation guidance, which is imperative to ensure consistent reporting.

## 6 Conclusion

This review identified that a diverse range of MATs exist internationally, with varying content, levels of detail, and guidance provided. No tools met the quality criteria to be recommended for use in their current form. Future research should prioritise seeking international consensus of acceptable content areas and recommendations included in MATs, to ensure a new tool is developed that is evidenced‐based, appropriate for the RAC population and informed by residents and stakeholders. More broadly, future efforts should focus on establishing nutritional targets for menus, developing best practice food and nutrition guidelines, and creating specific documents related to the mealtime experience. These future directions are essential to support the consistency of dietitian practice and, more importantly, to create meaningful changes to food and nutrition in RAC.

## Author Contributions

All authors approved the final version of the manuscript submitted for publication. All authors declare that the content of the manuscript has not been published elsewhere. **Karly Bartrim:** conceptualisation (lead), writing – original draft (lead), data collection (supporting) and formal analysis (lead), writing – review and editing (equal). **Alice Pashley:** conceptualisation (supporting), writing – original draft (supporting), data collection and formal analysis (supporting), writing – review and editing (equal). **Mikaela Wheeler:** conceptualisation (supporting), writing – original draft (supporting), data collection and formal analysis (supporting), writing – review and editing (equal). **Lily Chen:** conceptualisation (supporting), writing – original draft (supporting), data collection (supporting), writing – review and editing (equal). **Lisa Sossen:** conceptualisation (supporting), writing – original draft (supporting), data collection (supporting), writing – review and editing (equal). **Tracey Hancock:** data collection (supporting), writing – review and editing (equal). **Danielle Cave:** data collection (lead) and formal analysis (supporting), conceptualisation (supporting), writing – original draft (supporting), writing – review and editing (equal).

## Conflicts of Interest

The authors declare no conflicts of interest.

## Supporting information

Supplementary Table Agree.

## Data Availability

Data sharing not applicable to this article as no datasets were generated or analysed during the current study.
