# Supplementary material for: Menu Assessment Tools Used in Residential Aged Care: A Scoping Review of Content and Quality
Source: J Hum Nutr Diet. 2025 Jul 21;38(4):e70083. doi: 10.1111/jhn.70083 (PMC12280851; doi:10.1111/jhn.70083)
Supplement: Supplementary file 1 — Supplementary Table Agree. [file JHN-38-0-s001.docx]

**Table Supplementary 1: Appraisal of Guidelines for REsearch & Evaluation Instrument (AGREE II) Results for each Menu Assessment Tool**

| **Title of Document, Organisation/Author, Year of Publication and Reference** | **Domain 1: Scope & Purpose (%)** | **Domain 2: Stakeholder Involvement (%)** | **Domain 3: Rigour of Development (%)** | **Domain 4: Clarity of Presentation (%)** | **Domain 5: Applicability (%)** | **Domain 6: Editorial Independence (%)** | **Overall Quality of the Guideline (out of 7)** | **Recommended (Yes, Yes with modifications or No)*** |
| --- | --- | --- | --- | --- | --- | --- | --- | --- |
| Nutrition Standards for Meals and Menus, Queensland Health, 2022^30^ | 67 | 61 | 13 | 67 | 51 | 0 | 4 | Yes, with modifications |
| Apunipima Remote Residential Aged Care Menu Assessment Toolkit, Apunipima Cape York Health Council, 2015^35^ | 50 | 35 | 4 | 71 | 51 | 0 | 4 | No |
| Nutrition and Quality Food Standards for Adults in Victorian Public Hospitals and Residential Aged Care Services, Victorian Government Department of Health & Alfred Health Nutrition Department, 2022^31^ | 96 | 88 | 26 | 56 | 54 | 0 | 5 | Yes, with modifications |
| Best Practice Food and Nutrition Manual for Aged Care Homes, New South Wales Government, 2015^32^ | 89 | 78 | 21 | 74 | 64 | 4 | 5 | Yes, with modifications |
| Menu and Nutritional Standards for Public Health Facilities in South Australia, Department for Health and Wellbeing (Government of South Australia, 2020^33^ | 89 | 64 | 33 | 92 | 47 | 0 | 4 | No |
| Nutrition Standards for Adult Inpatients and Residential Aged Care Policy, Government of Western Australia, Country Health Service, 2020^34^ | 78 | 33 | 18 | 76 | 41 | 0 | 4 | No |
| Guidance on Food Served to Older People in Residential Care, Food Standards Agency, 2007^39^ | 85 | 24 | 5 | 49 | 30 | 0 | 3 | No |
| Eating Well for Older People, The Caroline Walker Trust, 2004^40^ | 93 | 69 | 29 | 72 | 72 | 6 | 5 | Yes, with modifications |
| Healthier and More Sustainable Catering. A Toolkit for Serving Food to Older People in Residential Care, Public Health England, 2017^38^ | 71 | 32 | 11 | 47 | 25 | 0 | 3 | No |
| Care Home Digest, Menu planning and food service guidelines for older adults living in care homes, British Dietetic Association Food Services Specialist Group and the Older People Specialist Group, 2024^15^ | 89 | 44 | 31 | 79 | 50 | 63 | 6 | Yes, with modifications |
| Nutritional guidelines and menu checklist for residential and nursing homes, Public Health Agency, 2014^43^ | 74 | 56 | 12 | 67 | 40 | 25 | 4 | No |
| Food and Nutrition in Care Homes for Older People, Welsh Government, 2019^41^ | 100 | 47 | 13 | 76 | 66 | 0 | 5 | No |
| Diet Manual for Long Term Care Residents, The Office of Health Care Quality, 2014^42^ | 28 | 18 | 4 | 38 | 18 | 0 | 2 | No |
| Best Practices for Nutrition, Food Service and Dining in Long Term Care Homes, Ontario Long Term Care Action Group, 2019^18^ | 36 | 19 | 10 | 54 | 52 | 0 | 3 | No |
| Menu Planning in Long Term Care, Dietitians of Canada, 2020^36^ | 83 | 33 | 23 | 44 | 54 | 0 | 4 | No |
| National Long Term Care Standards Project: Recommended Food & Nutrition Standards (Background Document), Dietitians of Canada (Brauer, P, Mardinlin-Vandewalle L & Whittington-Carter L), 2022^5^ | 76 | 56 | 45 | 81 | 46 | 0 | 5 | No |
| Audits and More: A Nutrition and Food Service Audit Manual for Adult Residential Care Facilities with 25 or more Persons with Care, Government of British Columbia, 2008^37^ | 72 | 64 | 23 | 67 | 74 | 0 | 5 | Yes, with modifications |

*Yes - Tools that scored >50% in all domains and scored four or more (out of seven) for overall quality, were recommended for use.

Yes, with modifications - Tools that scored >50% in four or more domains and scored four or more (out of seven) for overall quality, were recommended for use with modifications.

No - Tools that scored <50% in three or more domains and scored three or less (out of seven) for overall quality, were not recommended for use.
